# Editorial: Obesity, hyperglycemia, and pregnancy: from pathophysiology to clinical practice - volume II

**DOI:** 10.3389/fendo.2023.1283609

**Published:** 2023-09-07

**Authors:** Elena Succurro, Ester Vitacolonna

**Affiliations:** ^1^ Department of Medical and Surgical Sciences, University Magna Graecia of Catanzaro, Catanzaro, Italy; ^2^ Research Center for the Prevention and Treatment of Metabolic Diseases (CR METDIS), University Magna Graecia of Catanzaro, Catanzaro, Italy; ^3^ Department of Medicine and Aging, School of Medicine, and Health Sciences, “G. d’Annunzio” University, Chieti, Italy

**Keywords:** obesity, hyperglycemia, pregnancy, nutrition, pathophysiology

Adverse nutritional and genetic factors play a significant role in the pathogenesis of non-communicable diseases. Pregnancy represents a critical time window in which maternal metabolic disturbances, such as obesity and hyperglycemia, may result in short- and long-term complications for both the mother and the offspring.

There is strong evidence that even pre- and periconceptional parental insults can affect fetal development by activating specific key mechanisms of epigenetic reprogramming. Therefore, the study of trans-generational effects is a very promising field of research, as the knowledge of pathophysiological and clinical mechanisms induced by maternal obesity and hyperglycemia during pregnancy could shed light on the health of the next generation and provide useful preventive tools.

This Research Topic aims to provide insight into several facets of the connections between obesity, hyperglycemia, and pregnancy. The Research Topic encompasses four curated contributions, each covering different aspects.

Obesity negatively affects female fertility and is associated with infertility, abnormal menstruation, ovulation abnormalities (OA), polycystic ovary syndrome (PCOS), miscarriage, pregnancy complications, and neonatal congenital disabilities.

Long-term follow-up studies have shown that sleeve gastrectomy, which is the most commonly performed bariatric procedure worldwide, significantly increases postoperative patient pregnancy, live birth, and vaginal delivery rates and reduces pregnancy complications than women without surgical treatment. However, no research has identified a reliable indicator for predicting OA recovery after bariatric procedure.

Liu et al. in a prospective study performed in obese women who underwent sleeve gastrectomy for weight reduction, examined the prognostic usefulness of preoperative the luteinizing hormone (LH) to follicle-stimulating hormone (FSH) ratio (LFR). They found that LFR levels were the only component linked with ovulation abnormalities. Patients with a preoperative LFR of >182, who had a greater incidence of PCOS and more disrupted sex hormone levels, did not seem to regain ovulation despite achieving the same weight reduction 12 months after surgery. These findings underline the potential predictive role of preoperative sexual hormone levels, as expressed by LFR, on the fate of ovulation abnormalities following bariatric procedure.

Maternal obesity is also a key predictor for childhood obesity and neurodevelopmental delay in the offspring. This could be due to the shared genetic polymorphism between the obese mother and the child or changes of epigenome in the child. Medicinal plants are considered to be the safe and best option to treat maternal obesity, and at the same time, probiotic consumption during pregnancy provides beneficial effects for both the mother and the child. Current research has shown that Elateriospermum tapos (E. tapos) yoghurt is safe to consume and consists of many bioactive compounds that can exert an anti-obesity effect.

Naomi et al. investigated the role of E. tapos yoghurt in mitigating maternal obesity. In an experimental study performed on obese female Sprague Dawley rats in pregnancy, they found that supplementation of highest concentration of E. tapos yoghurt was associated with reduction in body weight and calorie intake and modulation of lipid levels, liver, and renal enzymes to a normal level. Moreover, in histological analysis, E. tapos yoghurt reversed the damage caused by high-fat diet in liver and colon, and the adipocytes’ hypertrophy in retroperitoneal white adipose tissue and visceral fat. This study provides useful insights into considering natural probiotics such as yoghurt as a therapeutic agent to treat maternal obesity.

Gestational diabetes mellitus (GDM) is a common endocrine disorder complicating up to 9-26% of pregnancies (1). GDM is also characterized by an acute inflammatory response mediated by circulating cytokines, which in turn are involved in the development of insulin resistance. This augmented maternal inflammatory environment may be closely associated to abnormal placental lipid metabolism, being a possible determinant of the increased incidence of adverse perinatal outcomes.

Visiedo et al. investigated the impact of key inflammatory mediators on the regulation of placental fatty acid metabolism from pregnancies complicated by GDM. They showed that women with GDM had elevated serum levels of IL- 6, TNF-a and leptin in the third trimester of pregnancy. These enhanced maternal cytokines levels were associated with an altered placental fatty acid metabolism, including a diminished placental fatty acid b‐oxidation capacity and an excess of free fatty acid accumulation in form of triglycerides. These findings highlight that an exacerbated proinflammatory environment in pregnancy would be closely related to the emergence of inadequate transport of essential lipids to the fetus and, consequently, to the onset of placental lipotoxic damage.

GDM is associated with adverse pregnancy outcomes, including increased rate of cesarean section, macrosomic infants, birth trauma and neonatal adverse outcomes (2). In long term perspective, GDM is associated with an increased risk of obesity, type 2 diabetes mellitus (T2DM) and cardiovascular diseases in mothers and their offspring (3). Maintaining normal glycaemic levels in pregnancy is essential to prevent adverse outcomes and long-term consequences (4–7).

Popova et al. aimed to investigate the effect of a mobile-based personalized recommendation system DiaCompanion I on glycaemic levels and pregnancy outcomes in women with GDM. Women with GDM will be randomized to 2 treatment groups: utilizing and not utilizing DiaCompanion I. The app provides women in the intervention group the resulting data-driven prognosis of 1-hour postprandial glucose levels, which are independent predictors of adverse maternal and perinatal outcomes and of T2DM (4). Moreover, the app also provides reminders and recommendations on diet and lifestyle to the participants of the intervention group. The primary outcome of this study is the percentage of postprandial capillary glucose values above target (>7.0 mmol/L). Secondary outcomes include the percentage of patients requiring insulin therapy during pregnancy, maternal and neonatal outcomes, glycaemic control, and the number of patient visits to endocrinologists and acceptance/satisfaction of the two strategies assessed using a questionnaire. The results of this study will provide insights into the efficacy of the app, including DiaCompanion I, in women with GDM in improving glycaemic levels and pregnancy outcomes.

In summary, this Research Topic has provided new insights into pathophysiological mechanisms and management strategies of GDM and potential predictors of infertility in women with obesity. This research could shed light on the health of the next generation and give useful preventive tools in the development of non-communicable diseases.

## Author contributions

EV: Writing – original draft, Writing – review & editing. ES: Writing – original draft, Writing – review & editing.
